# Genome-wide regulation of innate immunity by juvenile hormone and 20-hydroxyecdysone in the *Bombyx *fat body

**DOI:** 10.1186/1471-2164-11-549

**Published:** 2010-10-09

**Authors:** Ling Tian, Enen Guo, Yupu Diao, Shun Zhou, Qin Peng, Yang Cao, Erjun Ling, Sheng Li

**Affiliations:** 1 Key Laboratory of Developmental and Evolutionary Biology, Institute of Plant Physiology and Ecology, Shanghai Institutes for Biological Sciences, Chinese Academy of Sciences, Shanghai 200032, China; 2 Department of Sericulture Science, College of Animal Sciences, South China Agricultural University, Guangzhou 510642, China

## Abstract

**Background:**

Insect innate immunity can be affected by juvenile hormone (JH) and 20-hydroxyecdysone (20E), but how innate immunity is developmentally regulated by these two hormones in insects has not yet been elucidated. In the silkworm, *Bombyx mori*, JH and 20E levels are high during the final larval molt (4 M) but absent during the feeding stage of 5^th ^instar (5 F), while JH level is low and 20E level is high during the prepupal stage (PP). Fat body produces humoral response molecules and hence is considered as the major organ involved in innate immunity.

**Results:**

A genome-wide microarray analysis of *Bombyx *fat body isolated from 4 M, 5 F and PP uncovered a large number of differentially-expressed genes. Most notably, 6 antimicrobial peptide (AMP) genes were up-regulated at 4 M versus PP suggesting that *Bombyx *innate immunity is developmentally regulated by the two hormones. First, JH treatment dramatically increased AMP mRNA levels and activities. Furthermore, 20E treatment exhibited inhibitory effects on AMP mRNA levels and activities, and RNA interference of the 20E receptor *EcR*-*USP *had the opposite effects to 20E treatment.

**Conclusion:**

Taken together, we demonstrate that JH acts as an immune-activator while 20E inhibits innate immunity in the fat body during *Bombyx *postembryonic development.

## Background

Molting and metamorphosis in insects are coordinately regulated by the molting hormone 20-hydroxyecdysone (20E) and juvenile hormone (JH). Overall, 20E orchestrates the molting process, while JH determines the nature of the molt. In the presence of JH, 20E directs larval molting; while in the absence of JH, 20E directs larval-pupal-adult metamorphosis. In other words, JH antagonizes the 20E-induced physiological and developmental events to assure larval molting and to prevent larval-pupal-adult metamorphosis [1,2]. At the molecular level, for example, JH prevents 20E-induced programmed cell death (PCD) by suppressing the 20E-triggered transcriptional cascade and modulating mRNA levels of several caspase genes [3,4].

Insect fat body is the major organ involved in innate immunity, producing antimicrobial peptides (AMP) and other humoral response molecules [5]. AMP plays a central role in fighting against invading pathogens, which in turn up-regulate AMP gene expression via two distinct signaling pathways: the Toll pathway that is largely activated by fungi and Gram-positive bacteria and the Imd pathway that is mainly activated by Gram-negative bacteria [6]. In mammals, sex hormones and their nuclear receptors systematically regulate AMP production and thus innate immunity [7]. Nuclear receptors LXR, RXR, and PPAR also regulate AMP production [8]. However, little is known about hormonal regulation of innate immunity in insects apart from the fruitfly, *Drosophila melanogaster*. It has been suggested that 20E renders *Drosophila *mbn-2 cells and flies competent to induce AMP genes, such as *diptericin *and *drosomycin *[9-11]. Interestingly, *diptericin *expression could be induced by infection only after 3^rd ^instar larvae are mature enough to produce sufficient 20E [9]. Recently, it was shown that RNA interference (RNAi) of genes encoding the 20E receptor complex EcR-USP in *Drosophila *S2 cells prevented 20E-induced immune competence. In addition, JH III and JH agonist (JHA) strongly interfere with this 20E-dependent immune competence. This led to the suggestion that 20E acted as an immune-activator while JH an immune-suppressor [12]. In contrast, the genome-wide microarray study by Beckstead et al (2005) revealed that several AMP genes, including *cecropin C*, *attacin A*, *drosocin*, *drosomycin*, and *defensin*, were down-regulated by 20E in *EcR*-dependent manners. These authors assumed that 20E blocked innate immunity at the onset of metamorphosis [13]. The conflicting reports in *Drosophila *imply that 20E and JH regulate AMP mRNA expression in a complex manner and it is necessary to clarify this conflict in other insect species.

The silkworm, *Bombyx mori*, is one of the best models to study insect physiology and biochemistry. In the *Bombyx *genome, there are 35 AMPs belonging to 6 different types, namely Cecropins, Moricins, Gloverins, Attacins, Enbocins, and Lebocins. Among them, Cecropins, Moricins, Enbocins, and Lebocins have antibacterial activities against both Gram-positive and Gram-negative bacteria, while Gloverins and Attacins only have antibacterial activities against Gram-negative bacteria. Thus, the antibacterial spectrum of *Bombyx *shows much higher antibacterial activities against Gram-negative bacteria than those against Gram-positive bacteria [14].

Although some progress related to innate immunity has been made recently in *Bombyx*, very little is known about how innate immunity is developmentally regulated by insect hormones in this and other insect species. We have used a genome-wide microarray to analyze expression profiles of the fat body from the silkworm, *Bombyx mori*, during three developmental stages of animals displaying different 20E and JH levels. It revealed a large number of differentially-expressed genes, including 6 AMP genes. Hormone treatment and RNAi experiments demonstrate that JH acts as an immune-activator while 20E inhibits innate immunity in the fat body during *Bombyx *postembryonic development.

## Results

### Differentially-expressed genes revealed by microarray analyses

**Figure 1:**
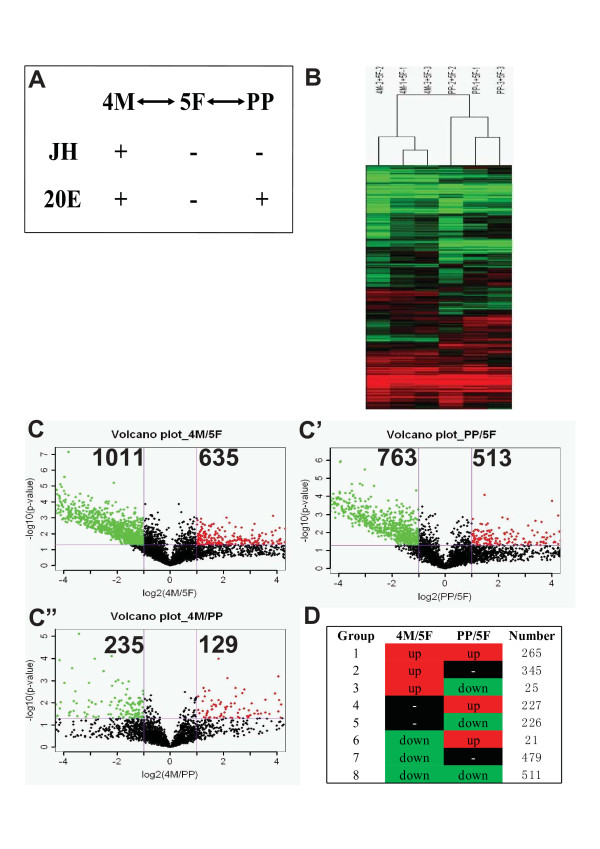
**Microarray analyses of the *Bombyx *fat body**. (A) Experimental design of microarray analyses. The dual-dye experiments were conducted between 4 M and 5 F as well as between PP and 5 F. Three biological replicates were used. (B) Hierarchical cluster analyses of three biological replicates. Green: down-regulation; red: up-regulation. (C-C") Volcano plots depicting estimated fold change (log_2_, x-axis) and statistical significance (-log_10 _P value, y-axis). Each point represents a gene, and colors correspond to the range of negative log_10 _P and log_2 _fold-change values. (D) Eight groups of differentially-expressed genes.

The genome map [15,16] and an oligonucleotide microarray [17] allowed us to investigate how 20E and JH developmentally regulate gene expression in the *Bombyx *fat body at the whole-genome scale. In this insect species, both 20E and JH levels are high during the final larval molt (4 M) but absent during the feeding stage of 5th instar (5 F), while 20E level is high and JH level is low at the prepupal stage (PP) [18,19]. Since both levels of 20E and JH vary dramatically from 4 M to 5 F to PP, we compared gene expression profiles between 4 M and 5 F as well as between 5 F and PP to understand how the two hormones regulate fat body physiological functions (Fig. 1A). Across all the 6 loading samples, 3300-3700 genes were reproducibly detected in each chip (Fig. 1B). We clustered the set of differentially-expressed genes by using hierarchical clustering with average linkage and chose cluster for further study by visual inspection. Hierarchical cluster (HC) results reflected that the three biological replicates between 4 M and 5 F or between PP and 5 F clustered together indicating the microarray analyses are repeatable (Fig. 1B). It appeared that the gene expression profiles varied dramatically from 4 M to 5 F and from 5 F to PP when analyzed using the volcano plots. The microarray results revealed that 1646 differentially-expressed genes were present between 4 M and 5 F (Fig. 1C) and 1276 between PP and 5 F (Fig. 1C'). Further statistical analysis revealed that 364 differentially-expressed genes were present between 4 M and PP (Fig. 1C"). Based on their expression profiles, the differentially-expressed genes were divided into 8 groups: genes in group 1 were up-regulated at both 4 M and PP in comparison with 5 F, genes in group 2 were up-regulated at 4 M versus 5 F while their expression levels had no differences between PP and 5 F, genes in group 3 were up- and down-regulated at 4 M and PP in comparison with 5 F, respectively, and so on (Fig. 1D). For example, many differentially-expressed genes in group 8 were involved in energy metabolism, including all genes in the glycolytic pathway. These genes were down-regulated by 20E at both 4 M and PP, when 20E levels are high [20].

### Differentially-expressed genes related to innate immunity

**Figure 2:**
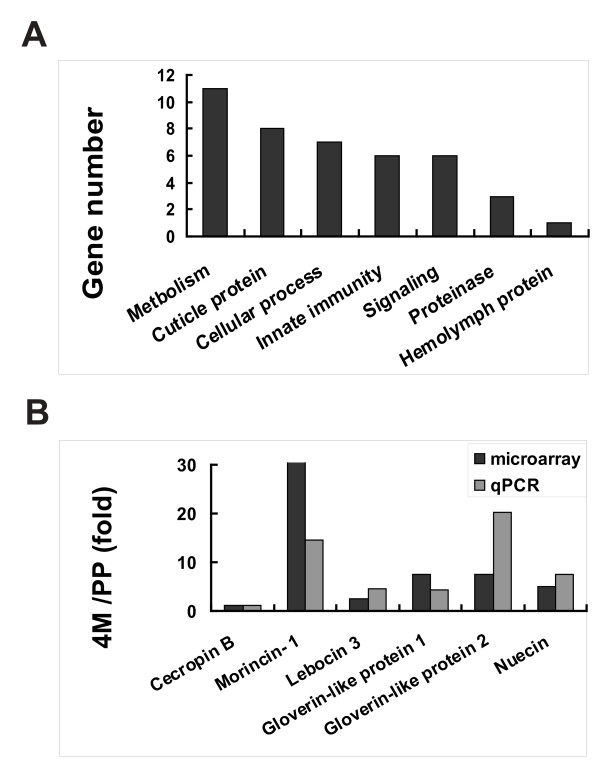
**Differentially-expressed genes among the three developmental stages**. (A) Classification of genes up-regulated at 4 M versus PP. (B) Changes of the transcriptional levels of the 6 AMP genes detected by microarray and validated by qPCR.

The set of differentially-expressed genes involved in innate immunity is particularly interesting. As shown in Figure 1C", 364 differentially-expressed genes were present between 4 M and PP. Among them, 129 genes were up-regulated at 4 M versus PP. Approximately 40% of these genes had unknown physiological functions and no significant BLAST hits. The classification of the function-defined genes was shown in Figure 2A. Most notably, 6 AMP genes, which were abundantly detected in each chip, were up-regulated at 4 M versus PP, including *cecropin B*, *moricin-1*, *lebocin-3*, 2 *gloverin-like proteins*, and *attacin *(a *nuecin *gene). Except *cecropin B*, the mRNA levels of the other 5 AMP genes were higher at 5 F in comparison with 4 M and PP. Changes of the transcriptional levels of the 6 AMP genes were validated by qPCR confirming the accuracy of microarray analyses (Fig. 2B). It is important to note that 20E levels are high at both 4 M and PP, while JH level is high at 4 M and low at PP. Thus, these data suggest that the 129 genes, particularly the 6 AMP genes, might be regulated by JH and 20E in a dynamic manner. In the following studies, we will focus on investigating how JH and 20E developmentally regulate AMP mRNA levels and activities in the fat body.

### Developmental changes of AMP activities

**Figure 3:**
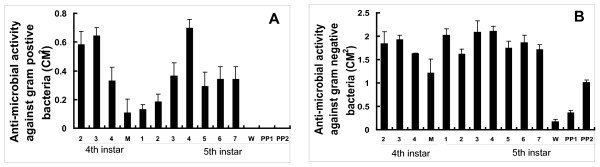
**Developmental changes of hemolymph AMP activities against Gram-positive bacteria (A: *Escherichia coli*) and Gram-negative bacteria (B: *Staphyloccocus aureus*)**. The paper count plates method was used for measuring AMP activities.

To understand whether and how JH and 20E regulate innate immunity during *Bombyx *postembryonic development, we first measured the developmental changes of AMP activities in the hemolymph, in comparison with the developmental changes of AMP mRNA levels. Antibacterial activities against both bacteria were high during the feeding stages of both 4^th ^and 5^th ^larval instars and low at both 4 M and PP, while antibacterial activities against both bacteria at 4 M were much higher than those at PP. In addition, antibacterial activity against Gram-negative bacteria was always higher than that against Gram-positive bacteria, which is in agreement with the *Bombyx *antibacterial spectrum [14]. Moreover, antibacterial activity against Gram-negative bacteria was undetectable at PP, whereas that against Gram-positive bacteria gradually increased from W to PP2 (Fig. 3A and 3B). Overall, the developmental changes of AMP activities are consistent with those of AMP mRNA levels measured by microarray and qPCR (Fig. 2B) strengthening the above hypothesis that JH and 20E developmentally regulate AMP mRNA levels and activities. Based on the developmental changes of JH and 20E titers as well as AMP mRNA levels and activities, we assume that JH plays a positive role in the regulation of innate immunity in the *Bombyx *fat body and the role of 20E is negative.

### Increase of AMP mRNA levels and activities by JH

**Figure 4:**
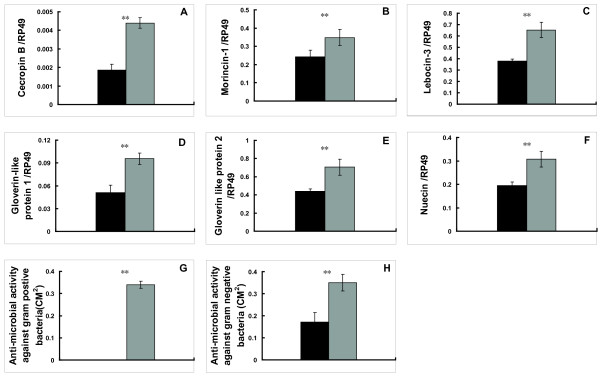
**Increases of AMP mRNA levels in the fat body (A-F) and AMP activities in the hemolymph (G-H) by JH treatment**. Black: control; gray: experimental. Ten animals were used for each group and 5 biological replicates were conducted in all the hormone treatment experiments. *: 0.01 < p < 0.05; **: p < 0.01.

It has been long believed that *status quo *action of JH is to antagonize 20E action [1,2]. To test the hypothesis that JH plays a positive role in the regulation of innate immunity, larvae at 12 hrs after EW were topically applied with JHA and then AMP mRNA levels and activities measured at 6 hours after JHA treatment. As predicted, JHA was able to effectively prevent the 20E-triggered transcriptional cascade, including the mRNA levels of the two 20E primary-response genes *E75B *and *Br-C *(Additional file 1), in the *Bombyx *fat body at this developmental stage. Remarkably, mRNA levels of all the 6 AMP genes were significantly increased by JHA treatment (Fig. 4A-F). Moreover, antibacterial activity against Gram-positive bacteria was increased from an undetectable level to a detectable level by JHA treatment, and antibacterial activity against Gram-negative bacteria was also significantly enhanced (Fig. 4G and 4H). The JHA treatment experiments demonstrate that JH is an immune-activator by increasing AMP mRNA levels and activities at 4 M.

### Decrease of AMP mRNA levels and activities by 20E

**Figure 5:**
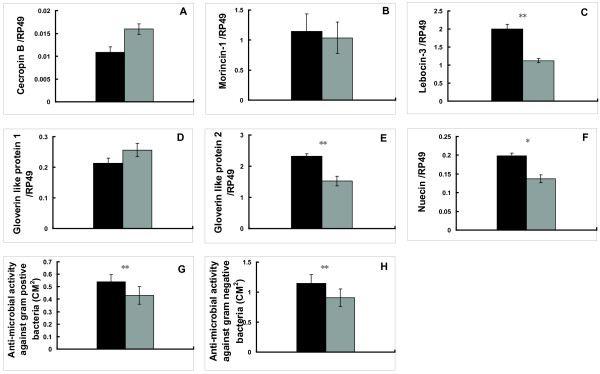
**Down-regulation of AMP mRNA levels (A-F) and activities (G-H) by 20E treatment**. Black: control; gray: experimental. Ten animals were used for each group and 5 biological replicates were conducted in all the hormone treatment experiments. *: 0.01 < p < 0.05; **: p < 0.01.

To test the hypothesis that 20E inhibits innate immunity during larval molts and the larval-pupal metamorphosis, we injected 20E into day 2 of 5^th ^instar larvae and measured AMP mRNA levels in the fat body and AMP activities in the hemolymph after 6 hours of 20E injection. As expected, mRNA levels of *E74B *and *Br-C *(Additional file 2) were significantly enhanced. The mRNA levels of 3 AMP genes (*lebocin-3*, *gloverin-like protein 2*, and *nuecin*) were significantly decreased and that of *morincin-1 *was slightly decreased. In contrast, 2 AMP genes (*cecropin B *and *gloverin-like protein 1*) were slightly up-regulated (Fig. 5. Meanwhile, AMP activities against both Gram-positive bacteria and Gram-negative bacteria were decreased by 20E injection (Fig. 5G and 5H). These experimental data were well agreement with the developmental changes of AMP mRNA levels and activities, which are low at 4 M and PP in the presence of 20E inhibition and high at 5 F in the absence of 20E inhibition. The 20E treatment experiments demonstrate that 20E has inhibitory effects on AMP mRNA levels and activities at both 4 M and PP and 20E acts as a general blocker for innate immunity at these stages.

### Increase of AMP mRNA levels and activities by *EcR-USP *RNAi

**Figure 6:**
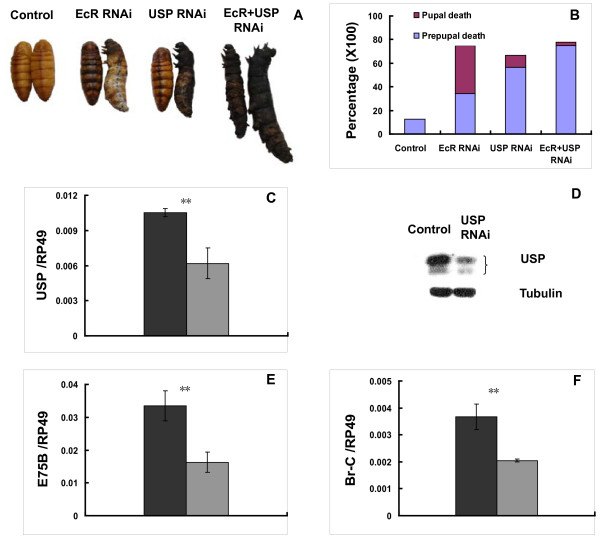
***EcR-USP *RNAi resulted in significant death phenotypes and disrupted 20E signaling**. (A) Typical prepupal death or pupal death phenotypes. (B) The ratio of prepupal death or pupal death. (C-D) *USP *mRNA (C) and USP protein (D) were decreased by *USP *RNAi. (E-F) mRNA levels of the two 20E primary-response genes *E75B *(E) and *Br-C *(F) were decreased. Black: control; gray: experimental. Thirty animals were used for each group and 3 biological replicates were conducted in the RNAi experiments. *: 0.01 < p < 0.05; **: p < 0.01.

20E acts via its receptor complex EcR-USP to trigger physiological and developmental events [21]. To verify if EcR-USP was responsible for 20E action in regulating innate immunity in *Bombyx *fat body, larvae at the initiation of EW were injected with *EcR *and/or *USP *dsRNA and then a series of assays were performed at 24 hours after RNAi treatment. First of all, RNAi resulted in significant prepupal or pupal death phenotypes. Some RNAi-treated animals failed to undergo larval-pupal metamorphosis and stopped as larval-pupal intermediates, whereas some others were arrested at the early pupal stage and exhibited a diapause-like phenotype (Fig. 6A). Treatment with both *EcR *and *USP *dsRNA resulted in significant (~75%) prepupal death. Injection of *USP *dsRNA alone resulted in ~60% prepupal death whereas *EcR *dsRNA alone caused ~40% prepupal death. In contrast, treatment with *EcR *dsRNA alone resulted in ~40% pupal death phenotype, with more limited pupal death observed with *USP *dsRNA alone (~10%) as well as both *EcR *and *USP *dsRNA (~5%) (Fig. 6B). We then measured if *EcR *RNAi and *USP *RNAi disrupted 20E signaling. Preliminary data showed that both *EcR *RNAi and *USP *RNAi had similar effects. In the following studies only experimental data for *USP *RNAi are presented because USP antibody is available and *USP *RNAi is a little more effective than *EcR *RNAi. *USP *mRNA was decreased to ~60% (Fig. 6C) while its protein decreased to ~30% (Fig. 6D) indicating the RNAi experiment was successful. Accordingly, mRNA levels of the two 20E primary-response genes *E75B *(Fig. 6E) and *Br-C *(Fig. 6F) were decreased about half. Considering some animals died earlier than 24 hours after *USP *RNAi treatment, the RNAi efficiency could be higher than detected.

**Figure 7:**
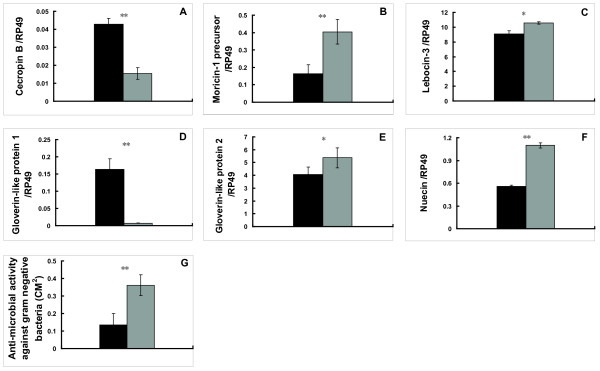
**Increases of AMP mRNA levels (A-F) and activities (G) by *USP *RNAi**. Black: control; gray: experimental. Thirty animals were used for each group and 3 biological replicates were conducted in the RNAi experiments. *: 0.01 < p < 0.05; **: p < 0.01.

AMP mRNA levels and activities were determined at 24 hours after RNAi treatment. The mRNA levels of 4 AMP genes (*morincin-1*, *gloverin-like protein 2*, *nuecin*, and *lebocin-3*) were significantly increased, while those of 2 AMP genes (*cecropin B *and *gloverin-like protein 1*) were significantly decreased (Fig. 7. Meanwhile, AMP activity against Gram-negative bacteria was increased by *USP *RNAi (Fig. 7G), but AMP activity against Gram-negative bacteria was still undetectable after *USP *RNAi. In summary, the effects on AMP mRNA levels and activities by *EcR-USP *RNAi (Fig. 7) are just opposite to 20E injection (Fig. 5) demonstrating that EcR-USP is responsible for 20E action in regulating AMP mRNA levels and activities.

## Discussion

A number of studies in *Drosophila *imply that 20E induces AMP mRNA expression and acts as an immune-activator [9,10,22], while JH acts as an immune-suppressor by antagonizing 20E signaling [12]. In contrast, genome-wide microarray studies in *Drosophila *strongly suggest that 20E-EcR-USP suppress AMP mRNA expression at the onset of metamorphosis [13]. The conflicting reports promoted us to study how 20E and JH coordinately regulate AMP mRNA expression in the *Bombyx *fat body.

The most important discovery in this paper is that JH is an immune-activator rather than an immune-suppressor in the *Bombyx *fat body. This conclusion is supported by a series of experiments. First, in our microarray and qPCR validation analyses, 6 AMP genes were up-regulated at 4 M (high JH level) versus PP (low JH level) in the *Bombyx *fat body (Fig. 2B). Again, developmental changes of AMP activities (Fig. 3) were in consistent with those of AMP mRNA levels (Fig. 2B). Moreover, JHA treatment increased mRNA levels of all the 6 AMP genes and antibacterial activities to both bacteria (Fig. 4).

**Figure 8:**
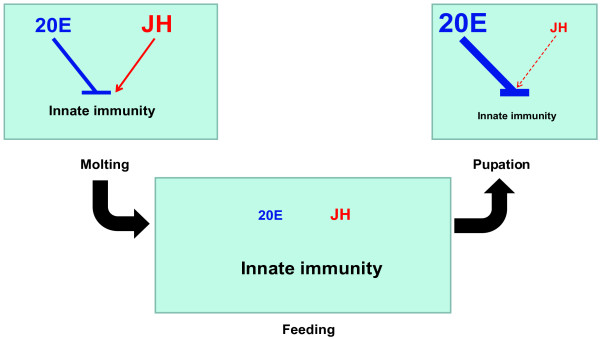
**A model showing developmental regulation of innate immunity by 20E and JH in the *Bombyx *fat body**. It demonstrates that 20E is a general blocker for innate immunity at 4 M and PP in the *Bombyx *fat body while JH antagonizes the action of 20E at 4 M. Text size conveys magnitude of treatment and response.

The second important discovery is that 20E is a general blocker for innate immunity in the *Bombyx *fat body. AMP mRNA levels and activities were low at both 4 M and PP (Fig. 2C and 3), when 20E levels are high. 20E down-regulated most AMP genes and decreased AMP activities (Fig. 5), while *EcR-USP *RNAi had opposite effects (Fig. 7). Based on our results in *Bombyx *and the microarray analyses in *Drosophila *[13], we conclude that 20E acts as a general blocker for innate immunity in insects via its receptor complex EcR-USP, during larval molts and the larval-pupal metamorphosis. Moreover, for the first time, we provide a clear diagram how JH and 20E dynamically regulate innate immunity in the fat body during *Bombyx *postembryonic development (Fig. 8). It is important to note that 20E are absent during the feeding stages, when AMP mRNA levels and activities are high due to the lack of 20E inhibition. During feeding, the animals are challenged by numerous pathogens in the diet, the high AMP mRNA levels and activities at these stages could be a basal protective mechanism. In comparison with the highly and rapidly inducible AMP mRNA levels and activities by invading pathogens [6,7], the hormonal regulation of innate immunity might be less important but could not be ignored.

In the larvae of many insect orders, particularly in Coleoptera, Orthoptera and Lepidoptera, the larval-pupal metamorphosis results from a low titer of JH and a high titer of 20E. In these insects, application of JH or JHA can prevent normal metamorphic events, resulting in a supernumerary larval molt. This is also the case in *Bombyx *[23,24]. For this reason, JH is referred to as the *status quo *hormone [1,25]. The major physiological function of JH during the larval molts is to antagonize 20E action and to prevent the 20E-induced physiological and developmental events [1,2]. For example, JH plays an important role by preventing 20E-induced PCD. At the molecular level, JH suppresses the 20E-triggered transcriptional cascade and down-regulates caspase genes [3,4]. However, because the JH receptor has never been identified, the JH signal transduction pathway remains unclear [2].

To our surprise, 20E did not down-regulate all the 6 AMP genes (Fig. 5), all of which were up-regulated by JH (Fig. 4) in *Bombyx*, although the fact that 20E up-regulates some AMP genes but down-regulates others is similar to *Drosophila *[12,13]. The biggest question that remains here is whether JH antagonizes the inhibitory effects of 20E to act as an immune-activator during larval molts. As expected, in the *Bombyx *fat body, JH was able to effectively prevent the 20E-triggered transcriptional cascade at EW (Supplementary Fig. 1). We assume the *status quo *action of JH is normally conserved in regulating innate immunity in the *Bombyx *fat body during the larval molts. To identify the JH receptor and to illustrate the JH signal transduction pathway will be necessary to understand the molecular mechanism how JH and 20E coordinately regulate innate immunity in *Bombyx *and other insect species.

## Conclusions

Combining the microarray, developmental changes, hormone treatments, and RNAi results together, we conclude that JH is an immune-activator rather than an immune-suppressor, while 20E inhibits innate immunity via its receptor complex EcR-USP. The two hormones dynamically regulate innate immunity in the fat body during *Bombyx *postembryonic development (Fig. 8).

## Methods

### Animals

*Bombyx *larvae (Nistari) were provided by The Sericultural Research Institute, Chinese Academy of Agricultural Sciences. They were reared with fresh mulberry leaves in the laboratory at 25°C under 14 hour light/10 hour dark cycles [26].

### Microarray analysis

The gene expression profiles in the *Bombyx *fat body were analyzed using silkworm genome 70-mer oligonucleotide microarray which covering over 23,000 *Bombyx *genes. The microarray was originally designed and constructed by Xia et al. (2007) and microarray analysis were performed and analyzed in the CapitalBio Corporation (Beijing, China). Fat body tissues were collected from larvae of three developmental stages: 4 M, 5 F, and PP and quickly frozen in liquid nitrogen. For 5 F, the same amount of fat body tissue was collected from day 1 to 7. Three biological replicates were used in this experiment. Total RNA was extracted from samples using Trizol reagent (Invitrogen, Gaithersburg, MD, USA), according to manufacturer's instructions. cDNA labeling with a fluorescent dye (Cy5 and Cy3-dCTP) was produced by Eberwine's linear RNA amplification method and subsequent enzymatic reaction using the procedure previously described [27], but with little modification by using CapitalBio cRNA Amplification and Labeling Kit (CapitalBio) for producing higher yields of labeled cDNA. After hybridization, the arrays were scanned with a confocal LuxScanTM scanner and the images obtained were then analyzed using LuxScanTM 3.0 software (Both from CapitalBio). Followed array data extracting, faint signals were removed if the intensities were below 400 units after background subtracted for both channels (Cy3 and Cy5). A space- and intensity-dependent normalization based on a LOWESS program was employed [28]. We identified significant differences in gene expression as those probes showing fold change > 2 and probewise P value < 0.05 [29]. The volcano plot method was used to estimate fold change The P values were calculated for the microarray data using the one sample or two sample t-test. Note that this does not control for false discovery rate and may overlook significant expression differences less than 2-fold, but we verified expression differences in the genes of primary interest using qPCR. For hierarchical analysis, we used average linkage clustering of the gene expression data (Cluster 3.0). Java Treeview (Stanford University, Stanford, Calif) was used for tree visualization. Gene functional categories were analyzed by Molecule Annotation System and KEGG http://www.kegg.com. The microarray data has been submitted to the Gene Expression Omnibus (GEO) under the accession number GSE23424.

### Hormone treatments

After a number of trials, day 2 of 5 F (48 hrs after 4 M) was chosen for 20E injection (Sigma Aldrich, USA) (3 μg/larva) and the controls were injected with the same volume of control solvent. At this stage, hemolymph 20E levels were low and the fat body was sensitive to 20E. Topical application of a JHA (methoprene, Dr. Ehrenstorfer GmbH, Germany, 15 μg/larva), was performed at 12 hrs after the initiation of EW. At this stage, 20E level just began to rise and the fat body was sensitive to JH. At 6 hours after 20E or JHA treatment, hemolymph was collected, and then larvae were sacrificed to dissect fat body tissues. Hemolymph samples were used for measurements of AMP activities and fat body samples were used for qPCR analysis. Ten animals were used for each group and 5 biological replicates were conducted.

### RNAi

Double-stranded RNA (dsRNA) of *Bombyx EcR *and *USP *[30] were generated using the T7 RiboMAX™ Express RNAi system (Promega, USA) according to the manufacturer's instruction. At the initiation of EW, each individual larva was injected with 5 μl of ddH2O, *EcR *dsRNA (5 μg), *USP *dsRNA (5 μg), or *EcR *(5 μg) and *USP *(5 μg) dsRNA. At this stage, the *Bombyx *larvae were sensitive to RNAi treatments. At 24 hrs after RNAi treatment, hemolymph was collected and the larvae were sacrificed for further measurements as described above. Thirty animals were used for each group and 3 biological replicates were conducted.

### Quantitative real-time PCR

Total RNA was extracted from larval fat body tissues of different developmental stages and used for quantitative real-time PCR (qPCR) analysis as previously described [25]. Primers used here and somewhere else in this paper are listed in Additional file 3.

### Measurements of AMP activities

The AMP activities of the hormone-treated silkworm cell-free plasma were measured using the paper count plates method as previously described [31]. Gram-negative and Gram-positive bacteria used in this study were *Escherichia coli *and *Staphyloccocus aureus*, respectively.

### Western blot analysis

AB11 USP-specific monoclonal antibody was provided by Dr. K.F. Kafatos (Harvard University). The Tubulin monoclonal antibody was purchased from Invitrogen. Western blot analysis was performed using standard procedures.

## Authors' contributions

LT and EG performed most of the experiments and analyzed the data. YD and QP carried out some experiments related to AMP activity. SZ did the Western blotting analysis. YC and EL helped design experiments and write the manuscript. SL designed the experiments, carried out some experiments, analyzed the data, wrote the paper, and coordinated the whole study. All authors approved the final manuscript.

## Supplementary Material

Additional file 1 **20E primary-response gene *E75B *and *Br-C *were down-regulated by JH treatment**. Click here for file

Additional file 2 **20E primary-response gene *E75B *and *Br-C *were up-regulated by 20E treatment**. Click here for file

Additional file 3 **A list of all PCR primers used in this paper**. Click here for file
