# Supplementary figures and images for: Genome-wide regulation of innate immunity by juvenile hormone and 20-hydroxyecdysone in the Bombyx fat body
Source: BMC Genomics. 2010 Oct 9;11:549. doi: 10.1186/1471-2164-11-549 (PMC3091698; doi:10.1186/1471-2164-11-549)

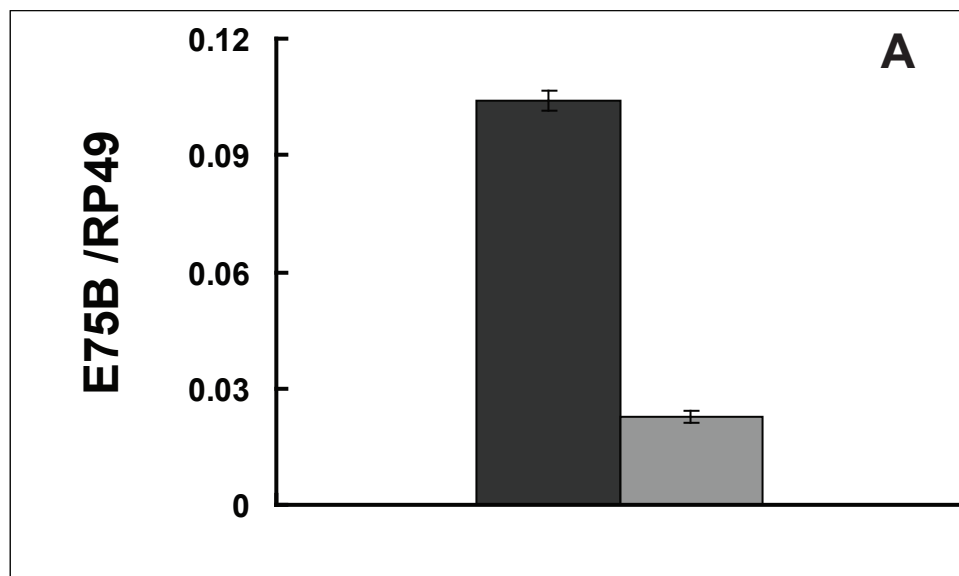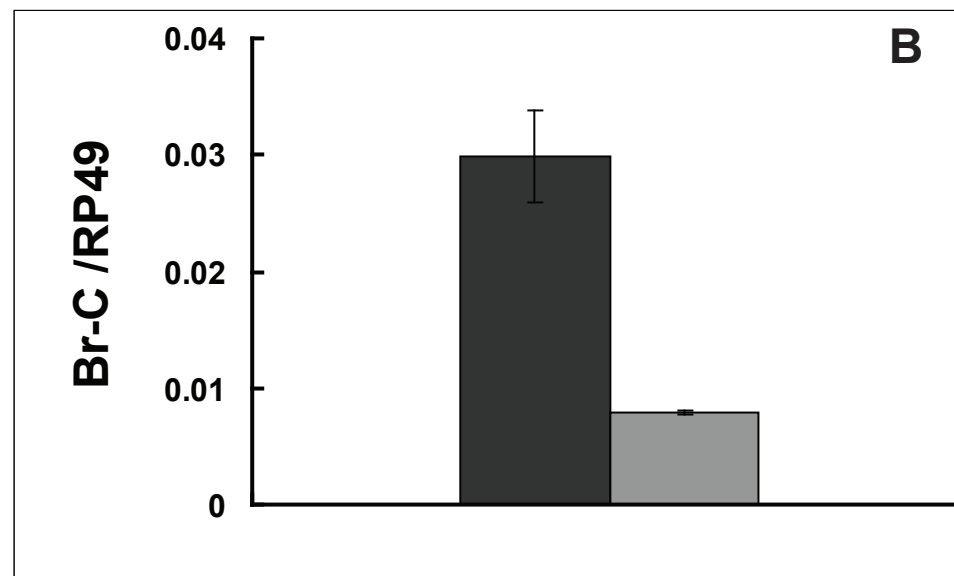

Supplement: Additional file 1 — 20E primary-response gene E75B and Br-C were down-regulated by JH treatment. [file 1471-2164-11-549-S1.PDF]

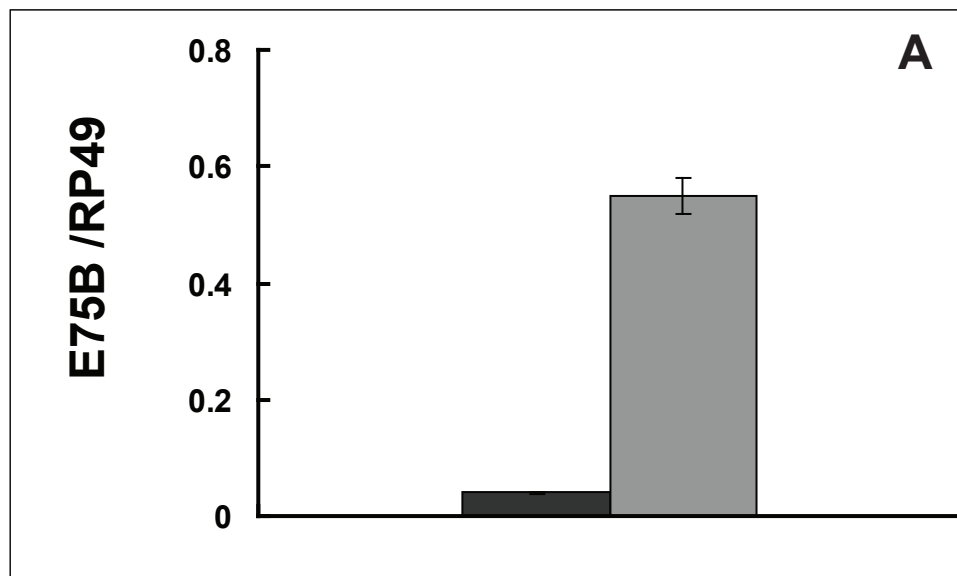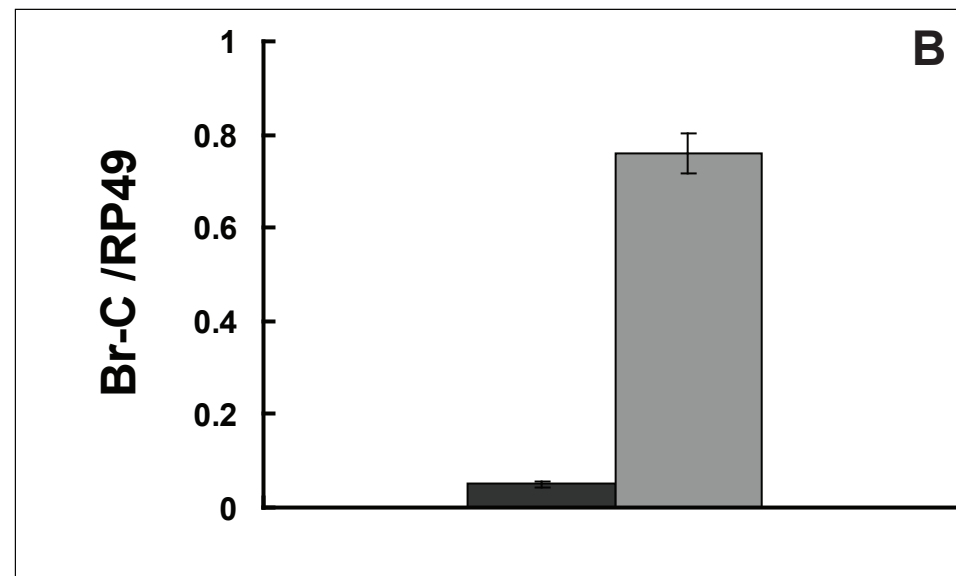

Supplement: Additional file 2 — 20E primary-response gene E75B and Br-C were up-regulated by 20E treatment. [file 1471-2164-11-549-S2.PDF]
