# Supplementary material for: Genome-wide regulation of innate immunity by juvenile hormone and 20-hydroxyecdysone in the Bombyx fat body
Source: BMC Genomics. 2010 Oct 9;11:549. doi: 10.1186/1471-2164-11-549 (PMC3091698; doi:10.1186/1471-2164-11-549)
Supplement: Additional file 3 — A list of all PCR primers used in this paper. [file 1471-2164-11-549-S3.PDF]

Primers for qPCR

Gene name	Genbank number	Primer sequence
RP49		S: CAGGCGGTTCAAGGGTCAATAC
		A: TGCTGGGCTCTTTCCACGA
Cecropin B	NM_001043927	S: GTGTTTCTGGTGTGTGCGA
		A: TATTTCCCGTGAGCGATG
Morincin 1	NM_001043364	S:TGTGGCAATGTCTCTGGTG
		A:GGCTGTACTGGCGATATTGA
Lebocin 3	NM_001044003	S:TCTGGTGCTGTTCTTTGCTC
		A:AGGAAGAATCGGATGGTCTG
Gloverin-like protein 1	NM_001099841;NM_001043465	S:TTGTCACTTGGGACAAGGAA
		A:TCCAGGCCCTAATACTCTGG
Gloverin-like protein 2	NM_001044218; NM_001043514	S:ACGCAGAAGTTTACGGACCT
		A:TTCCAAAGAGTCCATCATCG
Nuecin	NM_001043541	S:AGGCAAGGTCAACTTGTTCC
		A:GGTTGATGACGTCAGAGTGC
E75B	NM_001112610	S: CCCAAGATGACAAATTCACG
		A:TGCCCATTGAGACAGATGAT
Br-C	NM_001111334.1	S:AAGACGTGGCGTACACAGAC
		A:TCAGGAATGAGGACAAGCTG
EcR	NM_001043866	S: GCTGGTCTGATAACGGTGGCT
		A: CAAGGATTCCGGCGACATAAC
USP	NM_001044005	S:TCAAATAGGCAACAAACAGA
		A:CAGGAACTCCATAGACCG

Primers for dsRNA synthesis

EcR	NM_001043866	S: GGATCCTAATACGACTCACTATAGGGCGGTAGGAATGAGGC
		A: GGATCCTAATACGACTCACTATAGGTGGCGGAAGGGTAGAT
USP	NM_001044005	S: GGATCCTAATACGACTCACTATAGGCCCTAACCATCCCTTGA
		A: GGATCCTAATACGACTCACTATAGGTGAATCCGCAACTAACG
